# Relationship between childhood abuse and self-harm among transvestites and transgender women in Rio de Janeiro state 2019-2020

**DOI:** 10.1590/S2237-96222024v33e2024337.especial.en

**Published:** 2025-01-10

**Authors:** Davi Depret, Ricardo de Mattos Russo Rafael, Sonia Acioli, Mercedes Neto, Luciane de Souza Velasque, Virginia Maria Azevedo de Oliveira Knupp

**Affiliations:** 1 Universidade do Estado do Rio de Janeiro, Programa de Pós-graduação em Enfermagem, Rio de Janeiro, RJ, Brasil; 2 Universidade Federal do Estado do Rio de Janeiro, Departamento de Matemática e Estatística, Rio de Janeiro, RJ, Brasil; 3 Universidade Federal Fluminense, Departamento de Enfermagem, Rio das Ostras, RJ, Brasil

**Keywords:** Personas Transgénero, Comportamiento Autodestructivo, Violencia, Salud Mental, Políticas Públicas de Salud, Transgender People, Self-Destructive Behavior, Violence, Mental Health, Public Health Policy

## Abstract

**Objective:**

To analyze the relationship between childhood abuse and self-harm in a group of transvestites and transgender women from the state of Rio de Janeiro.

**Methods:**

This was a cross-sectional study conducted with 139 participants selected through convenience sampling between 2019 and 2020. A structured questionnaire was used for data collection. The odds ratio (OR) and 95% confidence intervals (95%CI) for suicidal ideation, suicide attempts, and self-harm were calculated using logistic regression.

**Results:**

Having experienced emotional abuse in childhood increased the suicide attempt (OR=9.00; 95%CI 1.13;71.34), having experienced psychological violence in childhood increased self-injurious behavior (OR=11.64; 95%CI 2.35;57.5), HIV infection increased suicidal ideation (OR=2.38; 95%CI 1.09;5.21).

**Conclusion:**

Childhood abuse, as well as the experience of stigmatizing diseases, increased the risk of self-harm among this population.

## Study contributions

**Main results:**

Childhood abuse had an impact on the mental health of transvestites and transgender women in adulthood. An increase in suicidal behavior and self-harm was observed among people who experienced childhood abuse.

**Implications for services:**

Healthcare services must be structurally and professionally equipped to meet the needs of this population, ensuring that institutional violence and revictimization do not occur.

**Perspectives:**

Future research on self-harm among transgender women and transvestites could contribute to the development of intersectoral public policies aimed at promoting mental health within this population, thereby reducing the incidence of this health condition.

## RESUMEN

**Objetivo:**

Analizar la relación entre las violencias sufridas en la infancia y la violencia autoinfligida en un grupo de travestis y mujeres transexuales del estado de Río de Janeiro.

**Métodos:**

Se trató de un estudio transversal realizado con 139 participantes seleccionadas mediante muestreo por conveniencia entre 2019 y 2020. Se utilizó un cuestionario estructurado para la recolección de datos. Se calcularon las razones de probabilidades (odds ratio, OR) y los intervalos de confianza del 95% (IC95%) para la ideación suicida, intentos de suicidio y autolesiones según las variables del estudio, a través de regresión logística.

**Resultados:**

Haber sufrido abuso emocional en la infancia aumentó los intentos de suicidio (OR=9,00; IC95% 1,13;71,34); haber sufrido violencia psicológica en la infancia incrementó el comportamiento autolesivo (OR=11,64; IC95% 2,35;57,5); la infección por VIH aumentó la ideación suicida (OR=2,38; IC95% 1,09;5,21).

**Conclusión:**

Las violencias sufridas en la infancia, así como la experiencia de enfermedades estigmatizantes, incrementaron el riesgo de violencia autoinfligida en esta población.

## INTRODUCTION

Childhood, as a distinct phase of life recognized in modern times, is a relatively recent phenomenon in which society acknowledges that children have specific rights. It is the responsibility of the State to ensure protection through legislation and public policies tailored to this population.^1^

Children are sometimes subjected to punishments and disciplinary actions that are considered pedagogical. These actions are driven by the cultural and collective belief that parents have absolute authority over their children’s bodies.^2^ Violence has intertwined with the upbringing processes in many families, seen as a tool for discipline and an instinctive response to disobedience and misconduct. ^2,3^

Violence against children and adolescents encompasses all contexts. This type of violence is inherently complex and multifaceted, influenced by a variety of factors. Most incidents are perpetrated by individuals close to and trusted by the young people.^2,4^

Globally, the failure of nations to protect their children allows violence to affect millions of young individuals daily. This compromises their quality of life and development, with some consequences only becoming evident in adulthood.^4,5^

When considering the experiences of children with non-normative identities, the additional stress they endure due to family rejection and exclusion becomes a predisposing factor for self-injurious behaviors. The early psychological burden faced by these individuals is associated with self-destructive behaviors, which may manifest in self-inflicted violence.^6^

The relationship between suicidal behavior among LGBTQIAPN+ people has been mapped. Higher rates of depression and prevalence of self-harm were found, especially among the transgender population, when compared to the general population.^7,8^

This study aimed to shed light on the underexplored topic of suicidal behavior among LGBTQIAPN+ individuals and to contribute to this investigation within the field of public health and gender and sexuality studies. The objective of this article was to analyze the relationship between childhood abuse and self-harm in a group of transvestites and transgender women in the state of Rio de Janeiro.

## METHODS

### Study design

This research is part of a broader set of studies that used the data from “EVAS (*Estudo sobre Violências e Autoavaliação em Saúde*): Study on Violence and Self-Assessment in Health”. This was a cross-sectional quantitative study involving a population of139 transvestites and transgender women.

### Setting

The research was conducted between June 2019 and March 2020 at the Laboratório de Pesquisa Clínica em DST e Aids, do Instituto Nacional de Infectologia Evandro Chagas, da Fundação Oswaldo Cruz, where the participants were monitored. Data collection involved in-person interviews. A structured questionnaire was used, completed virtually on a password-protected computer, in order to ensure participant confidentiality.

### Participants

The inclusion criteria adopted were: self-identification as transgender women (transvestites and transsexuals), age 18 years or older, and residence in the city of Rio de Janeiro or its metropolitan area. In order to increase adherence to the research, there were no exclusion criteria, apart from voluntary withdrawal.

Recruitment took place randomly at the reception of the do Instituto Nacional de Infectologia Evandro Chagas, during team visits, carried out by a trained team member. After explaining the study, participants who agreed to participate and met the criteria were taken to a room near the reception for data collection, where the informed consent form was read and a copy was provided to the participant.

### Variables

Among the 168 questions from the original research instrument, three questions three questions were selected for this study to assess self-harm. These questions mapped suicidal ideation (“In the past 12 months, have you thought about killing yourself?”), suicide attempts (“In the past 12 months, have you attempted to kill yourself?”) and self-injurious behavior (“In the past 12 months, have you cut, scratched, pierced, i.e, intentionally mutilated yourself?”). All questions offered “yes” or “no” as response options.

The outcome variable “self-harm” was defined in this study by a positive response to any of the three variables considered (ideation, attempt and self-injurious behavior) within the 12 months prior to the interview. These issues were addressed through direct questions about suicidal thoughts, suicidal attempts and signs of self-harm, such as self-mutilation, scratching and other acts of self-inflicted violence.

The exposure variables that characterized the participants’ profile included: gender identity (transvestite, transgender woman, other identities), sexual orientation, age group, race/skin color, residence in the city of Rio de Janeiro, living alone, marital status, years of education, formal employment, monthly income, religion, and HIV infection (self-reported). The exposure variables used to outline the violence experienced were: passability (the ability to navigate spaces without having their gender identity questioned) and discrimination based on appearance, gender identity (transvestite, trans woman, other identities) and sexual orientation (heterosexual, other orientations).

The variables used to map childhood abuse were: emotional abuse, physical abuse, sexual abuse, physical neglect and emotional neglect. Each variable was comprised of five specific questions from the Childhood Trauma Questionnaire (CTQ), adapted and cross-culturally validated for use in Brazil.^9^ In this study, a subscale divided the participant’s responses into four possible groups (none, mild to moderate, moderate to severe, severe to extremely severe).

According to the specific questions in the instrument used,^9^ emotional abuse was expressed by actions such as insulting and cursing the children and hearing the parents express a wish that the child had never been born. Sexual abuse included sexual touching and attempts, as well as threats involving such actions. Physical abuse was understood as being beaten to the point of leaving marks that required medical attention. Emotional neglect involved not feeling supported or loved, or being special. Physical neglect included having the presence of parents who were often under the influence of drugs or alcohol, leading to a lack of food or the inability to take the child to a doctor when necessary.

### Data sources and measurement

Univariate analyses, prevalence, and their respective 95% confidence intervals (95%CI) were performed. Bivariate analyses were performed using prevalence and odds ratios (OR) through logistic regression models, considering α values less than 0.05 to be statistically significant. Logistic regression was performed for dichotomous outcomes with variables having a p-value<0.30, following the probability of significance and effect measures of previous studies.^7,8^ The final logistic regression model was constructed.

The control of confounding variables was performed through statistical modeling using the manual backward elimination stepwise method.

### Statistical methods

The manual backward elimination stepwise method was applied, where variables with higher p-value were excluded until all remaining variables had p-values below 0.05, constructing a useful subset of predictors and arriving at the final model.

The database was initially created using the R Project for Statistical Computing software and was converted into a readable format for Stata SE 15 software (Stata Corp., College Station, United States), where data analyses were performed.

### Ethical aspects

The study complied with ethical guidelines for research involving human subjects and was approved by the Ethics Committee of the Universidade Federal do Estado do Rio de Janeiro under Opinion No. 3,182,376 of 04/03/2019, certificate of submission for ethical appraisal 07517419.0.0000.5285.^10^

## RESULTS

The sample consisted of 139 transvestites and transgender women aged 18 to 65 years. The majority identified as transgender woman (61.1%), heterosexual (95.0%), reasonably passable (67.6%), over the age of 35 years (51.8%), non-White (79.1%), with eight or more years of study (64.3%) and with some religious/spiritual affiliation (65.5%). Seventy-seven (55.4%) of them reported being HIV-positive at the time of the interview (Table 1).

**Table 1:** Prevalence of self-harm and sample characteristics of a group of transvestites and transgender women, Rio de Janeiro, Brazil, 2019-2020 (n=139)

Variables	n (%)
Self-injurious behavior	10 (7.2)
Suicide attempts	13 (9.3)
Suicidal ideation	40 (28.8)
**Gender identity**	
Others identities	17 (12.2)
Transvestite	37 (26.7)
Transgender woman	85 (61.1)
**Sexual orientation**	
Other orientations	7 (5.0)
Heterosexual	132 (95.0)
**Age group (years)**	
< 35	67 (48.2)
≥35	72 (51.8)
**Race/skin color**	
White	29 (20.9)
Non-White	110 (79.1)
**Resides in the municipality of Rio de Janeiro**	
No	37 (26.6)
Yes	102 (73.4)
**Lives alone**	
Yes	55 (39.9)
No	83 (60.1)
**Marital status**	
In a relationship	50 (36.0 )
Single	89 (64.0)
**Schooling (years)**	
<8	50 (36.0)
≥8	89 (64.0)
Religion	
No	48 (34.5)
Yes	91 (65.5)
**HIV infection status**	
Negative	62 (44.6)
Positive	77 (55.4)
**Works on the books**	
No	26 (18.7)
Yes	113 (81.3)
**Monthly income (BRL)**	
700-1,400	39 (28.1)
>1,400	40 (28.8)
<700	60 (43.1)
**Passability**	
High	45 (32.4)
Moderate	94 (67.6)
Emotional abuse	
None to minimum	55 (39.6)
Mild to extreme	84 (60.4)
**Physical abuse**	
None to minimum	59 (42.5)
Mild to extreme	80 (57.5)
**Sexual abuse**	
Mild to extreme	62 (44.6)
None to minimum	77 (55.4)
**Physical neglect**	
Mild to extreme	40 (28.8)
None to minimum	99 (71.2)
**Emotional neglect**	
None to minimum	55 ( 39.6)
Mild to extreme	84 (60.4)

Most participants (73.4%) lived in the municipality of Rio de Janeiro, did not live alone (60.1%) and were single (64.0%) at the time of the interview. Although the majority reported formal employment (81.3%), the predominant monthly income was up to BRL 700.00. It could be seen that 40 participants (28.8%) had experienced suicidal ideation, 13 participants (9.3%) had attempted suicide, and 10 participants (7.2%) had engaged in self-harm. Most participants had experienced some form of childhood abuse or neglect, including emotional abuse (60.4%), physical abuse (57.5%) and sexual abuse (55.4%), physical neglect (71.2%) and emotional neglect (60.4%) (Table 1).

In the bivariate analysis, when looking at outcomes individually, suicidal ideation was reported among participants who identified as transgender women (OR=0.54; 95%CI 0.23;1.23), had income between BRL700.00 and BRL1,400.00 (OR=2.83; 95%CI 1.02;7.86), lived in the city of Rio de Janeiro. (OR=0.56; 95%CI 0.25;1.25), considered themselves reasonably passable (OR=1.97; 95%CI 0.84;4.59), self-reported being HIV positive (OR=2.38; 95%CI 1.09;5.21), and had a history of child abuse: sexual abuse (OR=0.37; 95%CI 0.12;1.20), emotional abuse (OR=2.45; 95%CI 0.94;6.35), physical abuse (OR=0.25; 95%CI 0.05;1.18) and childhood neglect: physical neglect (OR= 2.80; 95%CI 0.69;11.33) and emotional neglect (OR=5.96; 95%CI 1.62;21.96)(Tables 2 and 3).

**Table 2:** Bivariate analysis of sociodemographic characteristics and self-harm among a group of transvestites and transgender women, Rio de Janeiro, Brazil, 2019-2020 (n=139)

**Variables**	**Suicidal ideation n (%)**	**OR (95%CI)**	**p-value**	**Suicide attempts n (%)**	**OR (95%CI)**	**p-value**	**Self-harm n (%)**	**OR (95%CI)**	**p- value**
Gender identity									
Transvestite	14 (37.8)	1.00		6 (16.2)	1.00		7 (19.4)	1.00	
Transgender woman	21 (24.7)	0.54 (0.23;1.23)	0.143	7 (8.2)	0.46 (0.14;1.49)	0.197	3 (3.5)	0.15 (0.04;0.62)	0.009
Other Identities	5 (29.4)	0.68 (0.20;2.36)	0.548	-	-	-	-		-
**Sexual orientation**									
Heterosexual	38 (28.8)	1.00		12 (9.1)	1.00		10 (7.63)	1.00	
Other orientations	2 (28.6)	0.99 (0.18;5.32)	0.990	1 (14.3)	1.67 (0.18;15.02)	<0.001	-	-	-
**Age group (years)**									
≥35	20 (27.8)	1.00		6 (8.3)	1.00		3 (4.2)	1.00	
<35	20 (29.8)	1.11 (0.53;2.31)	0.787	7 (10.4)	1.28 (0.41;4.03)	0.669	7 (10.4)	2.64 (0.65;10.68)	0.172
**Race/skin color**									
White	8 (27.6)	1.00		2 (6.9)	1.00		-	1.00	
Non-White	32 (29.1)	1.08 (0.43;2.68)	0.874	11 (10.0)	1.50 (0.31;7.18)	0.612	10 (9.1)	1.00 (0.05;1.91)	<0.001
**Resides in the municipality of Rio de Janeiro**									
No	14 (37.8)	1.00		4 (10.8)	1.00		3 (8.1)	1.00	
Yes	26 (25.5)	0.56 (0.25;1.25)	0.158	9 (8.8)	0.80 (0.23 ;2.77)	0.723	7 (6.9)	0.84 (0.21;3.45)	0.813
**Lives alone**									
No	24 (28.9)	1.00		10 (12.0)	1.00		6 (7.2)	1.00	
Yes	16 (29.1)	1.00 (0.48;2.14)	0.982	2 (3.6)	0.27 (0.06;1.31)	0.105	4 (7.2)	1.00 (0.27;3.74)	0.992
**Marital status**									
In a relationship	13 (26.0)	1.00		4 (8.0)	1.00		5 (10.0)	1.00	
Single	27 (30.3)	1.24 (0.57;2.69)	0.588	9 (10.1)	1.29 (0.38;4.44)	0.682	5 (5.7)	0.54 (0.15;1.97)	0.353
**Schooling (years)**									
≥8	26 (29.2)	1.00		9 (10.1)	1.00		7 (7.9)	1.00	
<8	14 (28.0)	0.94 (0.44;2.03)	0.879	4 (8.0)	0.77 (0.22;2.65)	0.682	3 (6.0)	0.74 (0.18 -2.99)	0.671
**Works on the books**									
No	8 (30.8)	1.00		2 (7.7)	1.00		2 (7.7)	1.00	
Yes	32 (28.3)	0.89 (0.35;2.25)	0.804	11 (9.7)	1.29 (0.27;6.22)	0.748	8 (7.1)	0.92 (0.18;4.63)	0.922
**Monthly income (BRL)**									
>1.400	6 (15.0)	1.00		2 (5.0)	1.00		1 (2.5)	1.00	
700-1.400	14 (35.9)	3.17 (1.07;9.41)	0.037	3 (7.7)	1.58 (0.25;10.03)	0.626	3 (7.7)	3.25 (0.32;32.68)	0.317
<700	20 (33.3)	2.83 (1.02;7.86)	0.045	8 (13.3)	2.92 (0.59;14.55)	0.190	6 (10.2)	4.41 (0.51;38.17)	0.177
**Passability**									
High	9 (20.1%)	1.00		3 (7.0%)	1.00		-	1.00	
Moderate	31 (33.2%)	1.97 (0.84;4.59)	0.117	10 (11.0%)	1.67 (0.43;6.38)	0.456	10 (11.0%)	1.00 (0.62;2.29)	<0.001
**Religion**									
No	14 (29.2)	1.00		6 (12.5%)	1.00		3 (6.2)	1.00	
Yes	26 (28.6)	0.97 (0.45;2.10)	0.941	7 (7.7%)	0.58 (0.18;1.84)	0.359	7 (7.8)	1.26 (0.31;5.13)	0.742
**HIV infection status**									
Negative	12 (19.3)	1.00		4 (6.4%)	1.00		3 (4.9)	1.00	
Positive	28 (36.4)	2.38 (1.09;5.21)	0.030	9 (11.7%)	1.91 (0.56;6.56)	0.298	7 (9.1)	1.93 (0.48;7.81)	0.355

**Table 3:** Bivariate analysis of childhood abuse and self-harm in a group of transvestites and transgender women, Rio de Janeiro, Brazil, 2019-2020 (n=139)

Variables	Suicidal ideation n (%)	OR (95%CI)	p-value	Suicide attempts n (%)	OR (95%CI)	p-value	Self-harm n (%)	OR (95%CI)	p-value
**Emotional abuse**									
None to minimum	12 (21.8)	1.00		1 (1.8)	1.00		1 (1.8)	1.00	
Mild to moderate	9 (26.5)	1.29 (0.48;3.49)	0.616	2 (5.9)	3.37 (0.29;38.71)	0.329	-	-	
Moderate to severe	6 (33.3)	1.79 (0.55;5.77)	0.329	2 (11.1)	6.75 (0.57;79.35)	0.129	3 (16.7)	10.8 (1.05;111.5)	0.046
Severe to extreme	13 (40.6)	2.45 (0.94;6.35)	0.065	8 (25.0)	18.00 (2.13;152.03)	0.008	6 (18.7)	12.46 (1.42;108.93)	0.023
**Physical abuse**									
None to minimum	19 (32.2)	1.00		2 (3.4)	1.00		1 (1.7)	1.00	
Mild to moderate	6 (26.1)	0.74 (0.25;2.19)	0.590	2 (8.7)	2.71 (0.36;20.52)	0.333	2 (9.1)	5.80 (0.50;67.46)	0.160
Moderate to severe	2 (10.5)	0.25 (0.05;1.18)	0.080	1 (5.3)	1.58 (0.13;18.50)	0.714	2 (10.5)	6.82 (0.58;79.91)	0.126
Severe to extreme	13 (34.2)	1.09 (0.46;2.60)	0.837	8 (21.0)	7.60 (1.51;38.07)	0.014	5 (13.2)	8.79 (0.98;78.45)	0.052
**Sexual abuse**									
None to minimum	23 (29.9)	1.00		7 (9.1)	1.00		4 (5.2)	1.00	
Mild to moderate	3 (50.0)	2.35 (0.44;12.51)	0.317	-	-	-	1 (16.7)	3.65 (0.34;39.09)	0.285
Moderate to severe	4 (13.8)	0.37 (0.12;1.20)	0.099	2 (6.9)	0.74 (0.14;3.79)	0.719	2 (7.1)	1.40 (0.24;8.12)	0.705
Severe to extreme	10 (37.0)	1.38 (0.55;3.47)	0.492	4 (14.8)	1.74 (0.47;6.48)	0.410	3 (11.1)	2.28 (0.48;10.92)	0.302
**Physical neglect**									
None to minimum	22 (22.2)	1.00		9 (9.0)	1.00		6 (6.1)	1.00	
Mild to moderate	11 (47.8)	3.21 (1.25;8.26)	0.016	1 (4.3)	0.45 (0.05;3.78)	0.466	2 (9.1)	1.55 (0.29;8.25)	0.607
Moderate to severe	4 (44.4)	2.80 (0.69;11.33)	0.149	2 (22.2)	2.86 (0.51;15.87)	0.230	1 (11.1)	1.94 (0.21;18.14)	0.562
Severe to extreme	3 (37.5)	2.10 (0.46;9.48)	0.335	1 (12.5)	1.43 (0.16;12.95)	0.751	1 (12.5)	2.21 (0.23;21.05)	0.489
**Emotional neglect**									
None to minimum	9 (16.4)	1.00		3 (5.4)	1.00		2 (3.6)	1.00	
Mild to moderate	7 (53.9)	5.96 (1.62;21.96)	0.007	1 (7.7)	1.44 (0.14;15.12)	0.759	1 (7.7)	2.21 (0.18;26.39)	0.531
Moderate to Severe	5 (33.3)	2.55 (0.70;9.28)	0.154	1 (6.7)	1.24 (0.12;12.84)	0.858	2 (13.3)	4.08 (0.52;31.72)	0.179
Severe to extreme	19 (33.9)	2.62 (1.06;6.48)	0.036	8 (14.3)	2.89 (0.72;11.52)	0.133	5 (9.1)	2.65 (0.49;14.29)	0.257

Suicide attempts were reported among participants who identified as transgender women (OR=0.46; 95%CI 0.14;-1.49), had an income between BRL 700.00 and BRL 1,400.00 (OR=2.92; 95%CI 0.59;-14.55), lived alone (OR=1.00; 95%CI 0.48;-2.14), self-reported being HIV-positive (OR=1.91; 95%CI 0.56;-6.56), and had a history of childhood abuse: emotional abuse (OR=18.00; 95%CI 2.13;-152.03), physical abuse (OR=7.60; 95%CI 1.51;-38.07), and childhood neglect: physical neglect (OR=2.86; 95%CI 0.51;15.87) and emotional neglect (OR=2.89; 95%CI 0.72;-11.52)(Tables 2 and 3).

Self-injurious behavior was reported among participants who identified as transgender women (OR=0.15; 95%CI 0.04;-0.62), had an income between BRL 700.00 and BRL 1,400.00 (OR=4.41; 95%CI 0.51;-38.17), were younger than 35 years (OR=1.11; 95%CI 0.53;-2.31), non-White (OR=1.08; 95%CI 0.43;-2.68), reasonably passable (OR=1.00; 95%CI 0.62;-2.29), and had a history of childhood abuse: sexual abuse (OR=3.65; 95%CI 0.34;-39.09), emotional abuse (OR=12.46; 95%CI 1.42;-108.93), physical abuse (OR=8.79; 95%CI 0.98;-78.45), and emotional neglect (OR=2.65; 95%CI 0.49;-14.29)(Tables 2 and 3).

HIV infection increased the likelihood of suicidal ideation by 2.4 times (95%CI 1.09;-5.21). Additionally, having experienced emotional abuse in childhood increased the likelihood of attempting suicide by 9 times (95%CI 1.13;-71.34) (Table 4).

**Table 4:** Multivariate logistic regression analysis of self-harm among a group of transvestites and transgender women, Rio de Janeiro, Brazil, 2019-2020 (n=139)

Variables	Ideation		Attempt		Self-injurious behavior	
	OR (95%CI)	p-value	OR (95%CI)	p-value	OR (95%CI)	p-value
**HIV infection**	2,4 (1,09;5,21)	0,030	-	-	-	-
**Emotional abuse**						
None to minimum	-	-	1,00	0,038	-	-
Mild to extreme	-	-	9,0 (1,13;71,34)		-	-
**Severe p sychological violence**	**-**	**-**	**-**	**-**	11,6 (2,35;57,5)	0,003

## DISCUSSION

The findings of this study align with data that highlighted a 47.25% prevalence of suicidal ideation and a 27.25% prevalence of suicide attempts among transgender women and transvestites.^11^

In this research, HIV-positive serology increased the likelihood of suicidal ideation. This corroborates global data, which emphasize the increased risk of suicidal behavior in clinical conditions with chronic outcomes, such as HIV.^12^ Despite advances in HIV treatment technologies related to the quality of life for people living with HIV, there remains a stigma burden associated with such a diagnosis.^13,14^

here was a significant association between HIV seropositivity and suicidal behavior.^15^ The lifetime prevalence of suicidal ideation was 25.2% in populations who had some clinical condition, which may have reached 35% in cases of comorbidities compared to 16.3% in the general population.^15^

Understanding the intersectional particularities of being a transvestite or transgender woman living with HIV, several challenges were identified, such as the lack of knowledge of the specific needs of HIV-positive transgender women.^16,17^ The lack of healthcare skills and barriers to comprehensive access to health services at all levels and prejudiced and stigmatizing attitudes from healthcare professionals in caring for these women contribute to their distancing from healthcare services.^17^ Such antagonistic behaviors represent non-compliance with established healthcare system standards and protocols, violating the right of HIV-positive transgender women to humane, respectful, and dignified care.^17^

There is no consensus in the literature about childhood transsexuality in, and this issue still remains controversial. The core of this discussion lies in the vulnerabilities reported by the participants of this study during their childhood, which were related to suicidal behavior in other stages of life ^18,19^

Another finding was the link between emotional abuse in childhood and an increased risk of developing suicidal behavior in adulthood. Emotional abuse is considered a predictor for various psychiatric disorders in adulthood, such as feelings of disconnection, rejection, impaired autonomy, loss of boundaries, hypervigilance, and inhibition of emotions.^20,21^ This can be explained by the decisive role that such traumatic events play in development of dysfunctional cognitive structures, negatively impacting crucial mental constructs necessary for healthy psychological development.^20,21^

Harmful events in the childhood of transgender children often serve as significant sources of formation and information for shaping the child’s self-concept. When this process is mediated by continuous episodes of emotional abuse, there is a greater risk of maladaptive development.^22^ Such patterns lead people with this history to seek out information congruent with their experiences, tending to perpetuate existing abusive patterns, which may manifest in self-abusive or even suicidal practices.^23^

It is essential to consider the Brazilian National Health System as a public and democratic health agency. This guarantees the exercise of citizenship as a popular and autonomous space, recognizes the influence of iniquities and incorporates mechanisms for the inclusion of new social subjects. It is also important to recognize and incorporate new social protection systems as promoters of social rights.^24^

In this context, the state assumes–or should assume–the role of guarantor of universal rights for all Brazilian citizens, regardless of their social, economic, racial, ethnic, sexual, or gender condition. The structuring of this system and its policies necessitates constant political mobilization to seek inclusion and social justice for all, tirelessly, across all social strata.^24^

At the structural and political level, the findings guide the need to provoke a debate on society as a locus of violence against transvestites and transgender people. These findings reinforce the urgency of establishing and consolidating actions aimed at promoting mental health and preventing suicide, as well as the early identification and intervention of its elements.^12^.^25^

As limitations of this study, the final number of participants, which was lower than expected due to the COVID-19 pandemic during the research period, stands out. This may lead to a final model that slightly diverges from the literature. It is also noted that the research setting may have introduced a sample selection bias regarding the prevalence of participants with HIV.

The relationship between gender identity, suicidal behavior and self-injurious behavior in transvestites and transgender women is scarcely addressed in the literature. There is a need for ongoing efforts to foster new investigative principles and, from the findings, to “transform” these data into tangible and promising realities. The goal is to envision a future for these women in which new frameworks of existence can be celebrated.

In conclusion, traumatic events that occurred in the childhood of transvestites and transgender women increased the likelihood of self-harm throughout their lives. Further studies are recommended, taking into consideration other realities to expand analytical possibilities, as well as the proposal of intersectoral public policies to address these issues.
