# Supplementary material for: Relationship between childhood abuse and self-harm among transvestites and transgender women in Rio de Janeiro state 2019-2020
Source: Epidemiol Serv Saude. 2025 Jan 10;33(spe1):e2024337. doi: 10.1590/S2237-96222024v33e2024337.especial.en (PMC11734651; doi:10.1590/S2237-96222024v33e2024337.especial.en)
Supplement: Translation

# Translation: Relação entre violências sofridas na infância e violência autoprovocada Relação entre violências sofridas na infância e violência autoprovocada entre travestis e mulheres transexuais do estado do Rio de Janeiro 2019-2020

**DOI:** 10.1590/S2237-96222024v33e2024337.especial.pt

**Authors:** Davi Depret, Ricardo de Mattos Russo Rafael, Sonia Acioli, Mercedes Neto, Luciane de Souza Velasque, Virginia Maria Azevedo de Oliveira Knupp

**Affiliations:** 1 Universidade do Estado do Rio de Janeiro, Programa de Pós-graduação em Enfermagem, Rio de Janeiro, RJ, Brasil; 2 Universidade Federal do Estado do Rio de Janeiro, Departamento de Matemática e Estatística, Rio de Janeiro, RJ, Brasil; 3 Universidade Federal Fluminense, Departamento de Enfermagem, Rio das Ostras, RJ, Brasil

**Keywords:** Pessoas Transgênero, Comportamento Autodestrutivo, Violência, Saúde Mental, Políticas Públicas de Saúde

## Abstract

**Objetivo:**

Analisar a relação entre as violências sofridas na infância e a violência autoprovocada em um grupo de travestis e mulheres transexuais do estado do Rio de Janeiro.

**Métodos:**

Tratou-se de estudo transversal realizado com 139 participantes selecionadas por meio de amostragem por conveniência entre 2019 e 2020. Foi utilizado questionário estruturado para a coleta de dados. Foi calculada a razão de chances (*odds ratio*, OR) e intervalo de confiança de 95% (IC_95_%) de ideação suicida, tentativa de suicídio e autoabuso pelas variáveis do estudo, por meio de regressão logística.

**Resultados:**

Ter sofrido abuso emocional na infância aumentou a tentativa de suicídio (OR=9,00; IC_95_% 1,13;71,34), ter sofrido violência psicológica na infância aumentou o comportamento autoabusivo (OR=11,64; IC_95_% 2,35;57,5), a infecção por HIV aumentou a ideação suicida (OR=2,38; IC_95_% 1,09;5,21).

**Conclusão:**

As violências sofridas na infância, bem como a vivência de doenças estigmatizantes, aumentaram o risco de violência autoprovocada entre essa população.

## Contribuições do estudo

**Principais resultados:**

As violências na infância impactaram a saúde mental de travestis e mulheres trans na idade adulta. Observou-se aumento de comportamento suicida e autoabuso nas pessoas que sofreram violência na infância.

**Implicações para os serviços:**

Os serviços de saúde devem estar preparados estrutural e profissionalmente para atender às demandas dessa população sem que ocorram violências institucionais tampouco a sua revitimização.

**Perspectivas:**

Investigações futuras sobre violência autoprovocada entre mulheres trans e travestis podem contribuir para a formulação de políticas públicas intersetoriais de promoção de saúde mental direcionadas a essa população, reduzindo a incidência desse agravo.

## INTRODUÇÃO

A infância, enquanto período da vida que se conhece na modernidade, é um fenômeno relativamente recente no qual a sociedade passou a reconhecer as crianças enquanto portadoras de direitos específicos. Cabe ao Estado a garantia de amparo através de legislações e políticas públicas próprias para esse público.^1^

A criança por vezes é objeto de punições e castigos considerados pedagógicos. Tais castigos são motivados pela arbietrariedade presente no imaginário coletivo e cultural de que os pais possuem total arbítrio sobre os corpos dos seus filhos.^2^ A violência se entremeou no processo formativo de muitas famílias entendida como um instrumento de responsabilização e resposta instintiva às indisciplinas e às desobediências.^2,3^

As violências praticadas contra crianças e adolescentes abarcam todos os contextos. Ratifica-se a natureza complexa e multifacetada desse tipo de violência, influenciada por diversos fatores. A maioria das ocorrências se dão por pessoas próximas e de confiança dos jovens.^2,4^

Em todo o mundo, a falha dos países em proteger suas crianças abre margem para que as violências atinjam milhões de jovens cotidianamente. Isso compromete a qualidade de vida e o desenvolvimento destes, com algumas reverberações que serão percebidas apenas na vida adulta.^4,5^

Considerando-se as experiências que permeiam as infâncias dissidentes, a carga de estresse adicional que essas crianças sofrem com a rejeição e a exclusão familiar se tornam fatores predisponentes para comportamentos autoabusivos. Devido ao potencial desgaste à saúde psíquica dessas pessoas antecipadamente, leva-se em consideração atitudes autodestrutivas, que podem se desdobrar em violências autoprovocadas.^6^

A relação do comportamento suicida entre pessoas LGBTQIAPN+ foi mapeada. Apontaram-se maiores taxas de depressão e prevalência de violência autoprovocada, sobretudo na população trans, quando comparada com a população em geral.^7,8^

Houve a tentativa de desvelar a relação do comportamento suicida entre pessoas LGBTQIAPN+, temática que é pouco explorada, e contribuir para tal investigação tanto no campo da saúde coletiva como nos estudos de gênero e sexualidade. Este artigo objetivou analisar a relação entre as violências sofridas na infância e a violência autoprovocada em grupo de travestis e mulheres transexuais do estado do Rio de Janeiro.

## MÉTODOS

### Delineamento

Esta pesquisa foi inserida no conjunto de pesquisas que utilizaram os dados do “EVAS: Estudo sobre Violências e Autoavaliação em Saúde”. Tratou-se de estudo transversal quantitativo, cuja população foi de 139 mulheres transexuais e travestis. 

### Contexto

A pesquisa foi realizada entre junho de 2019 e março de 2020 no Laboratório de Pesquisa Clínica em DST e Aids, do Instituto Nacional de Infectologia Evandro Chagas, da Fundação Oswaldo Cruz, onde as participantes eram acompanhadas. Para a coleta dos dados, foram realizadas entrevistas presenciais. Utilizou-se questionário estruturado, preenchido de maneira virtual em um computador com senha, visando garantir o sigilo quanto à identificação da participante. 

### Participantes

Os critérios de inclusão adotados foram: autoidentificação como mulheres transexuais (travestis e transexuais), idade maior ou igual a 18 anos e residência no município do Rio de Janeiro ou na sua área metropolitana. Visando à maior adesão à pesquisa, não houve critérios de exclusão, a não ser a saída voluntária de qualquer participante. 

O recrutamento se deu de maneira aleatória na recepção do Instituto Nacional de Infectologia Evandro Chagas, durante os dias de ida da equipe, por um membro previamente treinado. Após a explicação do estudo, em caso de aceite e inclusão nos critérios, conduziu-se a participante até uma sala próxima à recepção para a coleta de dados, onde foi lido o termo de consentimento livre e esclarecido e entregue a cópia deste para a participante.

### Variáveis 

Entre as 168 questões do instrumento de pesquisa original, três perguntas foram utilizadas para o recorte desta pesquisa, configurando o panorama das violências autoprovocadas. Mapearam-se a ideação suicida (“Nos últimos 12 meses, você pensou em se matar?”), a tentativa de suicídio (“Nos últimos 12 meses, você tentou se matar?”) e o comportamento autoabusivo (“Nos últimos 12 meses, você se cortou, se arranhou, perfurou, ou seja, se mutilou propositalmente?”). Para todas as perguntas, as possibilidades de resposta eram “sim” ou “não”.

A variável desfecho “violência autoprovocada” foi definida nesta pesquisa pela resposta positiva para qualquer uma das três variáveis consideradas (ideação, tentativa e autoabuso) ocorridas nos últimos 12 meses prévios à entrevista. As questões foram abordadas através de perguntas diretas acerca de pensamentos suicidas, tentativas suicidas e sinais de autoabuso, como automutilação, arranhaduras e demais ações de violência autoinflingida.

As variáveis de exposição que compuseram o perfil das participantes foram: identidade de gênero (travesti, mulher trans, outras identidades), orientação afetiva sexual, faixa etária, raça/cor da pele, moradia no município do Rio de Janeiro, se morava sozinha, situação conjugal, tempo de estudo, vínculo formal de trabalho, renda mensal, religião, infecção por HIV (autorreferido). As variáveis de exposição utilizadas para diagramar as violências sofridas foram: passabilidade (possibilidade de adentrar espaços sem ter sua identidade de gênero questionada) e discriminação por aparência, identidade de gênero (travesti, mulher trans, outras identidades) e orientação afetiva sexual (heterossexual, outras orientações). 

As variáveis que mapearam as violências na infância foram: abuso emocional, abuso físico, abuso sexual, negligência física e negligência emocional. Cada variável foi composta por cinco questões específicas do instrumento “Questionário sobre Traumas na Infância” (QUESI, originalmente denominado Childhood Trauma Questionnaire – CTQ), adaptado e validado transculturalmente para uso no Brasil.^9^ Neste estudo foi utilizada subescala que dividiu as respostas de cada participante em quatro grupos possíveis (não a mínimo, leve a moderado, moderado a severo, severo a grave).

De acordo com as questões específicas do instrumento utilizado,^9^ o abuso emocional foi expresso por ações como insultar e xingar as crianças e ouvir dos pais a exteriorização do desejo que o filho nunca tivesse nascido. O abuso sexual se manifestou por toques sexuais e tentativas, bem como ameaças envolvendo tais ações. Compreendeu-se como abuso físico ser espancado e ficar com marcas a ponto de precisar ir ao médico. A negligência emocional envolveu não sentir que tinha fonte de apoio e suporte e não se sentir amado ou especial. A negligência física abarcava ter a presença de pais drogados ou alcoolizados boa parte do tempo, a ponto de faltar comida ou de não ser possível levá-las ao médico se necessário.

### Fontes de dados e mensuração

Foram realizadas as análises univariadas, as prevalências e os respectivos intervalos de confiança de 95% (IC_95_%). As análises bivariadas foram através das prevalências e as razões de chance (*odds ratio*, OR) por meio de modelos de regressão logística, considerando estatisticamente significante os valores de α inferiores a 0,05. Realizou-se a regressão logística para os desfechos dicotômicos com as variáveis de p-valor<0,30, seguindo a probabilidade de significância e medidas de efeito de estudos anteriores.^7,8^ O modelo final de regressão logística foi construído.

O controle das variáveis confundidoras foi feito através da modelagem estatística com a realização do método de passo a passo manual de eliminação regressiva.

### Métodos estatísticos

Aplicou-se o método de passo a passo manual de eliminação regressiva, no qual as variáveis com maior p-valor foram excluídas até que todas as variáveis presentes tivessem p-valor inferior a 0,05, construindo um subconjunto útil de preditores e chegando ao modelo final. 

O banco de dados foi inicialmente criado no software R Project for Statistical Computing e foi convertido para o formato de leitura do *software* Stata SE 15 (Stata Corp., College Station, Estados Unidos), onde foram realizadas as análises de dados.

### Aspectos éticos

O estudo obedeceu aos preceitos éticos de pesquisas envolvendo seres humanos e foi aprovado pelo Comitê de Ética em Pesquisa da Universidade Federal do Estado do Rio de Janeiro sob o número do Parecer nº 3.182.376 de 4/03/2019, certificado de apresentação para apreciação ética 07517419.0.0000.5285.^10^

## RESULTADOS

A amostra foi composta por 139 travestis e mulheres transexuais entre 18 e 65 anos. A maioria se apresentou como mulher transexual (61,1%), heterossexual (95,0%), razoavelmente passável (67,6%), na faixa etária acima de 35 anos (51,8%), não branca (79,1%), com oito anos ou mais de estudo (64,3%) e com alguma afiliação religiosa/espiritual (65,5%). Setenta e sete (55,4%) delas relataram *status* sorológico positivo de HIV no momento da entrevista (Tabela 1).

**Tabela 1:** Prevalência de violência autoprovocada e características amostrais de grupo de travestis e mulheres transexuais, Rio de Janeiro, 2019-2020 (n=139)

Variáveis	n (%)
Comportamento autoabusivo	10 (7,2)
Tentativa de suicídio	13 (9,3)
Ideação suicida	40 (28,8)
**Identidade de gênero**	
Outras identidades	17 (12,2)
Travesti	37 (26,7)
Mulher trans	85 (61,1)
**Orientação afetiva sexual**	
Outras orientações	7 (5,0)
Heterossexual	132 (95,0)
**Faixa etária (anos)**	
<35	67 (48,2)
≥35	72 (51,8)
**Raça/cor da pele**	
Branca	29 (20,9)
Não branca	110 (79,1)
**Reside no município do Rio de Janeiro**	
Não	37 (26,6)
Sim	102 (73,4)
**Mora sozinha**	
Sim	55 (39,9)
Não	83 (60,1)
**Situação conjugal**	
Comprometida	50 (36,0)
Solteira	89 (64,0)
**Tempo de estudo (anos)**	
<8	50 (36,0)
≥8	89 (64,0)
**Religião**	
Não	48 (34,5)
Sim	91 (65,5)
**Infecção por HIV**	
Negativo	62 (44,6)
Positivo	77 (55,4)
**Trabalha com carteira assinada**	
Não	26 (18,7)
Sim	113 (81,3)
**Renda mensal (R$)**	
700-1.400	39 (28,1)
>1.400	40 (28,8)
<700	60 (43,1)
**Passabilidade**	
Muita	45 (32,4)
Razoável	94 (67,6)
**Abuso emocional**	
Não a mínimo	55 (39,6)
Leve a extremo	84 (60,4)
**Abuso físico**	
Não a mínimo	59 (42,5)
Leve a extremo	80 (57,5)
**Abuso sexual**	
Leve a extremo	62 (44,6)
Não a mínimo	77 (55,4)
**Negligência física**	
Leve a extremo	40 (28,8)
Não a mínimo	99 (71,2)
**Negligência emocional**	
Não a mínimo	55 (39,6)
Leve a extremo	84 (60,4)

A maioria delas (73,4%) morava no município do Rio de Janeiro, não morava sozinha (60,1%) e estava solteira (64,0%) no momento da entrevista. Embora a maioria tenha declarado trabalhar com vínculo formal (81,3%), a renda mensal predominante foi de até R$ 700,00. Observou-se que 40 participantes (28,8%) tiveram ideação suicida, 13 participantes (9,3%) colocaram em prática alguma tentativa de suicídio e 10 (7,2%) se automutilaram propositalmente. A maioria das participantes sofreu algum tipo de agressão ou ataque na infância, compreendendo desde abuso emocional (60,4%), abuso físico (57,5%) e abuso sexual (55,4%) à negligência física (71,2%) e negligência emocional (60,4%) (Tabela 1). 

Na análise bivariada, de maneira isolada entre os desfechos, houve relato de ideação suicida entre as participantes que se identificavam como mulher trans (OR=0,54; IC_95_% 0,23;1,23), com renda entre R$ 700,00 e R$ 1.400,00 (OR=2,83; IC_95_% 1,02;7,86), que moravam no município do Rio de Janeiro (OR=0,56; IC_95_% 0,25;1,25), com passabilidade autoavaliada razoável (OR=1,97; IC_95_% 0,84;4,59), com autorrelato de infecção por HIV (OR=2,38; IC_95_% 1,09;5,21), com histórico de abusos infantis: abuso sexual (OR=0,37; IC_95_% 0,12;1,20), abuso emocional (OR=2,45; IC_95_% 0,94;6,35), abuso físico (OR=0,25; IC_95_% 0,05;1,18) e negligências na infância: negligência física (OR=2,80; IC_95_% 0,69;11,33) e negligência emocional (OR=5,96; IC_95_% 1,62;21,96) (Tabelas 2 e 3).

Houve relato de tentativa de suicídio entre as participantes que se identificavam como mulher trans (OR=0,46; IC_95_% 0,14;1,49), com renda entre R$ 700,00 e R$ 1.400,00 (OR=2,92; IC_95_% 0,59;14,55), que moravam sozinhas (OR=1,00; IC_95_% 0,48;2,14), com autorrelato de infecção por HIV (OR=1,91; IC_95_% 0,56;6,56), com histórico de abusos infantis: abuso emocional (OR=18,00; IC_95_% 2,13;152,03), abuso físico (OR=7,60; IC_95_% 1,51;38,07) e negligências na infância: negligência física (OR=2,86; IC_95_% 0,51;15,87) e negligência emocional (OR=2,89; IC_95_% 0,72;11,52)(Tabelas 2 e 3).

O comportamento autoabusivo foi relatado entre as participantes que se identificavam como mulher trans (OR=0,15 IC_95_% 0,04;0,62), com renda entre R$ 700,00 e R$ 1.400,00 (OR=4,41; IC_95_% 0,51-38,17), com até 35 anos (OR=1,11; IC_95_% 0,53;2,31), não-brancas (OR=1,08; IC_95_% 0,43;2,68), com passabilidade autoavaliada razoável (OR=1,00; IC_95_% 0,62;2,29),com histórico de abusos infantis: abuso sexual (OR=3,65; IC_95_% 0,34;39,09), abuso emocional (OR=12,46; IC_95_% 1,42;108,93), abuso físico (OR=8,79; IC_95_% 0,98;78,45) e negligência emocional na infância. (OR=2,65; IC_95_% 0,49;14,29)(Tabelas 2 e 3).

**Tabela 2:** Análise bivariada entre características sociodemográficas e violência autoprovocada em grupo de travestis e mulheres transexuais, Rio de Janeiro, 2019-2020 (n=139)

**Variáveis**	**Ideação suicida n (%)**	**OR (IC**_95_ **% )**	**p-valor**	**Tentativa de suicidio n (%)**	**OR (IC**_95_ **% )**	**p-valor**	**Autoabuso n (%)**	**OR (IC**_95_ **% )**	**p- valor**
Identidade de gênero									
Travesti	14 (37,8)	1,00		6 (16,2)	1,00		7 (19,4)	1,00	
Mulher trans	21 (24,7)	0,54 (0,23;1,23)	0,143	7 (8,2)	0,46 (0,14;1,49)	0,197	3 (3,5)	0,15 (0,04;0,62)	0,009
Outras identidades	5 (29,4)	0,68 (0,20;2,36)	0,548	-	-	-	-		-
**Orientação afetiva sexual**									
Heterossexual	38 (28,8)	1,00		12 (9,1)	1,00		10 (7,63)	1,00	
Outras orientações	2 (28,6)	0,99 (0,18;5,32)	0,990	1 (14,3)	1,67 (0,18;15,02)	<0,001	-	-	-
**Faixa etária (anos)**									
≥35	20 (27,8)	1,00		6 (8,3)	1,00		3 (4,2)	1,00	
<35	20 (29,8)	1,11 (0,53;2,31)	0,787	7 (10,4)	1,28 (0,41;4,03)	0,669	7 (10,4)	2,64 (0,65;10,68)	0,172
**Raça/cor da pele**									
Branca	8 (27,6)	1,00		2 (6,9)	1,00		-	1,00	
Não branca	32 (29,1)	1,08 (0,43;2,68)	0,874	11 (10,0)	1,50 (0,31;7,18)	0,612	10 (9,1)	1,00 (0,05;1,91)	<0,001
**Mora no município do Rio de Janeiro**									
Não	14 (37,8)	1,00		4 (10,8)	1,00		3 (8,1)	1,00	
Sim	26 (25,5)	0,56 (0,25;1,25)	0,158	9 (8,8)	0,80 (0,23 ;2,77)	0,723	7 (6,9)	0,84 (0,21;3,45)	0,813
**Mora sozinha**									
Não	24 (28,9)	1,00		10 (12,0)	1,00		6 (7,2)	1,00	
Sim	16 (29,1)	1,00 (0,48;2,14)	0,982	2 (3,6)	0,27 (0,06;1,31)	0,105	4 (7,2)	1,00 (0,27;3,74)	0,992
**Situação conjugal**									
Comprometida	13 (26,0)	1,00		4 (8,0)	1,00		5 (10,0)	1,00	
Solteira	27 (30,3)	1,24 (0,57;2,69)	0,588	9 (10,1)	1,29 (0,38;4,44)	0,682	5 (5,7)	0,54 (0,15;1,97)	0,353
**Tempo de estudo (anos)**									
≥8	26 (29,2)	1,00		9 (10,1)	1,00		7 (7,9)	1,00	
<8	14 (28,0)	0,94 (0,44;2,03)	0,879	4 (8,0)	0,77 (0,22;2,65)	0,682	3 (6,0)	0,74 (0,18 -2,99)	0,671
**Trabalha com carteira assinada**									
Não	8 (30,8)	1,00		2 (7,7)	1,00		2 (7,7)	1,00	
Sim	32 (28,3)	0,89 (0,35;2,25)	0,804	11 (9,7)	1,29 (0,27;6,22)	0,748	8 (7,1)	0,92 (0,18;4,63)	0,922
**Renda mensal (R$)**									
>1.400	6 (15,0)	1,00		2 (5,0)	1,00		1 (2,5)	1,00	
700-1.400	14 (35,9)	3,17 (1,07;9,41)	0,037	3 (7,7)	1,58 (0,25;10,03)	0,626	3 (7,7)	3,25 (0,32;32,68)	0,317
<700	20 (33,3)	2,83 (1,02;7,86)	0,045	8 (13,3)	2,92 (0,59;14,55)	0,190	6 (10,2)	4,41 (0,51;38,17)	0,177
**Passabilidade**									
Muita	9 (20,1%)	1,00		3 (7,0%)	1,00		-	1,00	
Razoável	31 (33,2%)	1,97 (0,84;4,59)	0,117	10 (11,0%)	1,67 (0,43;6,38)	0,456	10 (11,0%)	1,00 (0,62;2,29)	<0,001
**Religião**									
Não	14 (29,2)	1,00		6 (12,5%)	1,00		3 (6,2)	1,00	
Sim	26 (28,6)	0,97 (0,45;2,10)	0,941	7 (7,7%)	0,58 (0,18;1,84)	0,359	7 (7,8)	1,26 (0,31;5,13)	0,742
**Infecção por HIV**									
Negativo	12 (19,3)	1,00		4 (6,4%)	1,00		3 (4,9)	1,00	
Positivo	28 (36,4)	2,38 (1,09;5,21)	0,030	9 (11,7%)	1,91 (0,56;6,56)	0,298	7 (9,1)	1,93 (0,48;7,81)	0,355

**Tabela 3:** Análise bivariada entre violências sofridas na infância e violência autoprovocada em grupo de travestis e mulheres transexuais, Rio de Janeiro, 2019-2020 (n=139)

Variáveis	Ideação suicida n (%)	OR (IC_95_ **%**)	p-valor	Tentativa de suicidio n (%)	OR (IC_95_ **%**)	p-valor	Autoabuso n (%)	OR (IC_95_ **%**)	p- valor
**Abuso emocional**									
Não a mínimo	12 (21,8)	1,00		1 (1,8)	1,00		1 (1,8)	1,00	
Leve a moderado	9 (26,5)	1,29 (0,48;3,49)	0,616	2 (5,9)	3,37 (0,29;38,71)	0,329	-	-	
Moderado a severo	6 (33,3)	1,79 (0,55;5,77)	0,329	2 (11,1)	6,75 (0,57;79,35)	0,129	3 (16,7)	10,8 (1,05;111,5)	0,046
Severo a extremo	13 (40,6)	2,45 (0,94;6,35)	0,065	8 (25,0)	18,00 (2,13;152,03)	0,008	6 (18,7)	12,46 (1,42;108,93)	0,023
**Abuso físico**									
Não a mínimo	19 (32,2)	1,00		2 (3,4)	1,00		1 (1,7)	1,00	
Leve a moderado	6 (26,1)	0,74 (0,25;2,19)	0,590	2 (8,7)	2,71 (0,36;20,52)	0,333	2 (9,1)	5,80 (0,50;67,46)	0,160
Moderado a severo	2 (10,5)	0,25 (0,05;1,18)	0,080	1 (5,3)	1,58 (0,13;18,50)	0,714	2 (10,5)	6,82 (0,58;79,91)	0,126
Severo a extremo	13 (34,2)	1,09 (0,46;2,60)	0,837	8 (21,0)	7,60 (1,51;38,07)	0,014	5 (13,2)	8,79 (0,98;78,45)	0,052
**Abuso sexual**									
Não a mínimo	23 (29,9)	1,00		7 (9,1)	1,00		4 (5,2)	1,00	
Leve a moderado	3 (50,0)	2,35 (0,44;12,51)	0,317	-	-	-	1 (16,7)	3,65 (0,34;39,09)	0,285
Moderado a severo	4 (13,8)	0,37 (0,12;1,20)	0,099	2 (6,9)	0,74 (0,14;3,79)	0,719	2 (7,1)	1,40 (0,24;8,12)	0,705
Severo a extremo	10 (37,0)	1,38 (0,55;3,47)	0,492	4 (14,8)	1,74 (0,47;6,48)	0,410	3 (11,1)	2,28 (0,48;10,92)	0,302
**Negligência física**									
Não a mínimo	22 (22,2)	1,00		9 (9,0)	1,00		6 (6,1)	1,00	
Leve a moderado	11 (47,8)	3,21 (1,25;8,26)	0,016	1 (4,3)	0,45 (0,05;3,78)	0,466	2 (9,1)	1,55 (0,29;8,25)	0,607
Moderado a severo	4 (44,4)	2,80 (0,69;11,33)	0,149	2 (22,2)	2,86 (0,51;15,87)	0,230	1 (11,1)	1,94 (0,21;18,14)	0,562
Severo a extremo	3 (37,5)	2,10 (0,46;9,48)	0,335	1 (12,5)	1,43 (0,16;12,95)	0,751	1 (12,5)	2,21 (0,23;21,05)	0,489
**Negligência emocional**									
Não a mínimo	9 (16,4)	1,00		3 (5,4)	1,00		2 (3,6)	1,00	
Leve a moderado	7 (53,9)	5,96 (1,62;21,96)	0,007	1 (7,7)	1,44 (0,14;15,12)	0,759	1 (7,7)	2,21 (0,18;26,39)	0,531
Moderado a severo	5 (33,3)	2,55 (0,70;9,28)	0,154	1 (6,7)	1,24 (0,12;12,84)	0,858	2 (13,3)	4,08 (0,52;31,72)	0,179
Severo a extremo	19 (33,9)	2,62 (1,06;6,48)	0,036	8 (14,3)	2,89 (0,72;11,52)	0,133	5 (9,1)	2,65 (0,49;14,29)	0,257

A infecção por HIV aumentou em 2,4 (IC_95_% 1,09; 5,21) vezes a prevalência de ter ideação suicida. Observou-se também ter sofrido algum tipo de abuso emocional na infância aumentou em 9 (IC_95_% 1,13;71,34) vezes a chance de tentativa de suicídio (Tabela 4). 

**Tabela 4:** Análise multivariada por regressão logística da violência autoprovocada em grupo de travestis e mulheres transexuais. Rio de Janeiro, 2019-2020 (n=139)

Variáveis	Ideação		Tentativa		Comportamento autoabusivo	
	OR (IC _95_ % )	p-valor	OR (IC _95_ % )	p-valor	OR (IC _95_ % )	p-valor
**Infecção por HIV**	2,4 (1,09;5,21)	0,030	-	-	-	-
**Abuso emocional**						
Não a mínimo	-	-	1,00	0,038	-	-
Leve ao extremo	-	-	9,0 (1,13;71,34)		-	-
**Violência psicológica grave**	-	-	-	-	11,6 (2,35;57,5)	0,003

## DISCUSSÃO

Os resultados gerados neste estudo se aproximaram de dados que evidenciaram a prevalência de ideação suicida de 47,25% e tentativa de suicídio de 27,25% entre travestis e mulheres trans.^11^

Nesta pesquisa, a sorologia positiva de HIV aumentou a ideação suicida. Isso corroborou os dados mundiais, os quais salientaram que houve aumento do risco de comportamento suicida em quadros de acometimentos clínicos cujo desfecho é crônico, como o caso do HIV.^12^ Apesar dos avanços nas tecnologias do tratamento do HIV relacionados à qualidade de vida das pessoas com HIV, ainda há o fardo que estigmatiza tal diagnóstico.^13,14^

Houve associação significativa entre soropositividade e comportamento suicida.^15^ A prevalência de ideação suicida ao longo da vida foi 25,2% em populações que possuíam alguma condição clínica, podendo ter chegado a 35% em casos de comorbidades *versus* 16,3% na população geral.^15^

Entendendo-se as particularidades interseccionais de ser travesti ou mulher trans com HIV, alguns dificultadores foram apontados, a exemplo do desconhecimento das necessidades específicas das mulheres trans soropositivas.^16,17^ Falta de habilidades assistenciais e barreiras de acesso integral aos serviços de saúde em todos os seus níveis e atitudes preconceituosas e estigmatizantes por parte dos profissionais de saúde no cuidado dessas mulheres contribuem para seu afastamento dos serviços de saúde.^17^ Tais comportamentos antagônicos representaram o descumprimento de normas e protocolos estabelecidos pelos sistemas de saúde, violando o direito à saúde das mulheres trans HIV positivas ao serviço humano, respeitoso e digno.^17^

Não existe consenso na literatura acerca da transexualidade na infância, e essa temática ainda parece ser polêmica. O ponto de sustentação dessa discussão esteve justamente nas vulnerabilidades relatadas pelas participantes desta pesquisa durante suas infâncias e que tiveram relação com comportamento suicida em outras fases da vida.^18,19^

Outro resultado encontrado foi a relação de abusos emocionais sofridos na infância e do aumento do risco de desenvolvimento do comportamento suicida na vida adulta. Sobre essa relação, considerou-se que o abuso emocional pode ser preditor para diversos transtornos psiquiátricos na vida adulta, como sentimentos de desconexão, rejeição, autonomia prejudicada, perda de limites, supervigilância e inibição de sentimentos.^20,21^ Isso pode ser explicado pelo papel decisivo que tais eventos traumáticos exercem no desenvolvimento de estruturas cognitivas disfuncionais, impactando negativamente aparatos psíquicos importantes para uma constituição mental saudável.^20,21^

Eventos nocivos ocorridos na infância das crianças trans acabam sendo utilizados como importantes fontes de formação e informação para estruturar o autoconceito da criança. Quando esse processo ocorre mediado por episódios contínuos de abuso emocional, existe maior risco de que se constituam de forma desadaptativa.^22^ Tais padrões levariam as pessoas com esse histórico a buscarem informações que lhes são congruentes, tendendo a perpetuar o padrão abusivo existente, o que pode se manifestar em práticas autoabusivas ou até mesmo suicidas.^23^

É necessário pensar o Sistema Único de Saúde enquanto instância pública e democrática de saúde. Este garante o exercício da cidadania como espaço popular e autônomo, reconhece a influência das iniquidades e incorpora mecanismos de inclusão de novos sujeitos sociais. Faz-se também importante reconhecer e incorporar novos sistemas de proteção social na qualidade de promotores dos direitos sociais.^24^

O Estado, nesse lugar, assume – ou deveria assumir – papel de garantidor de direitos universais a todos os cidadãos brasileiros, independentemente de sua condição social, econômica, racial, étnica, sexual e de gênero. A estruturação desse sistema e de suas políticas traz a necessidade constante de mobilizações políticas para que se busque inclusão e justiça social para todos, de maneira incansável, em todos as camadas sociais.^24^

No nível estrutural e político, os resultados encontrados orientam a provocação de debate do entendimento da sociedade enquanto lócus de violência contra travestis e pessoas trans. Esses resultados reforçam a urgência de se constituir e consolidar ações voltadas para a promoção da saúde mental e prevenção do suicídio, bem como a identificação e intervenção precoces de seus elementos.^12,25^

Como limitações deste estudo, destaca-se o número final de participantes abaixo do esperado, tendo em vista a pandemia de covid-19 no transcorrer da pesquisa. Isso pode levar a um modelo final que destoa levemente da literatura. Pontua-se que o cenário de pesquisa pode ter gerado um viés de seleção da amostra quanto à prevalência de participantes com HIV.

Essa relação entre identidade de gênero, comportamento suicida e comportamento autoabusivo em travestis e mulheres transexuais é pouco abordada na literatura. Evidencia-se a necessidade de empreitadas recorrentes para que novos princípios investigativos surjam e para que, a partir dos resultados encontrados, esses dados se “trans-formem” em realidades tangíveis e exultantes. A finalidade é de que essas mulheres visem ao futuro no qual se possa celebrar novas conjunturas de existência.

Em conclusão, os eventos traumáticos ocorridos na infância de travestis e mulheres trans aumentou a chance de violência autoprovocada ao longo da vida. Recomenda-se a realização de mais estudos que levem em consideração outras realidades as quais expandam possibilidades analíticas, bem como proposição de políticas públicas intersetoriais de enfrentamento.
